# Aquaporins: relevance to cerebrospinal fluid physiology and therapeutic potential in hydrocephalus

**DOI:** 10.1186/1743-8454-7-15

**Published:** 2010-09-22

**Authors:** Brian K Owler, Tom Pitham, Dongwei Wang

**Affiliations:** 1 Kids Neurosurgical Research Unit, Institute of Neuroscience and Muscle Research, Kids Research Institute, Children's Hospital at Westmead, Hawkesbury Rd, Westmead NSW 2145, Australia; 2 TY Nelson Department of Neurology and Neurosurgery, Children's Hospital at Westmead, Hawkesbury Rd, Westmead NSW 2145, Australia; 3 Discipline of Child Health and Paediatrics, Children's Hospital at Westmead Clinical School, University of Sydney, Hawkesbury Rd, Westmead NSW 2145, Australia

## Abstract

The discovery of a family of membrane water channel proteins called aquaporins, and the finding that aquaporin 1 was located in the choroid plexus, has prompted interest in the role of aquaporins in cerebrospinal fluid (CSF) production and consequently hydrocephalus. While the role of aquaporin 1 in choroidal CSF production has been demonstrated, the relevance of aquaporin 1 to the pathophysiology of hydrocephalus remains debated. This has been further hampered by the lack of a non-toxic specific pharmacological blocking agent for aquaporin 1. In recent times aquaporin 4, the most abundant aquaporin within the brain itself, which has also been shown to have a role in brain water physiology and relevance to brain oedema in trauma and tumours, has become an alternative focus of attention for hydrocephalus research. This review summarises current knowledge and concepts in relation to aquaporins, specifically aquaporin 1 and 4, and hydrocephalus. It also examines the relevance of aquaporins as potential therapeutic targets in hydrocephalus and other CSF circulation disorders.

## Introduction

Aquaporins are a family of integral membrane proteins that function as water channels. The existence of such water channels had been postulated for some time as the passage of water across certain membranes is too rapid to be explained on the basis of diffusion through plasma membranes [1]. The identification of AQP1[2], initially named CHIP28 [3], was later followed by the identification of 12 other aquaporins. Aquaporins are distributed widely throughout the body but notably in the kidney, red blood cells, lung and secretory epithelia such as the salivary glands [1].

**Figure 1:**
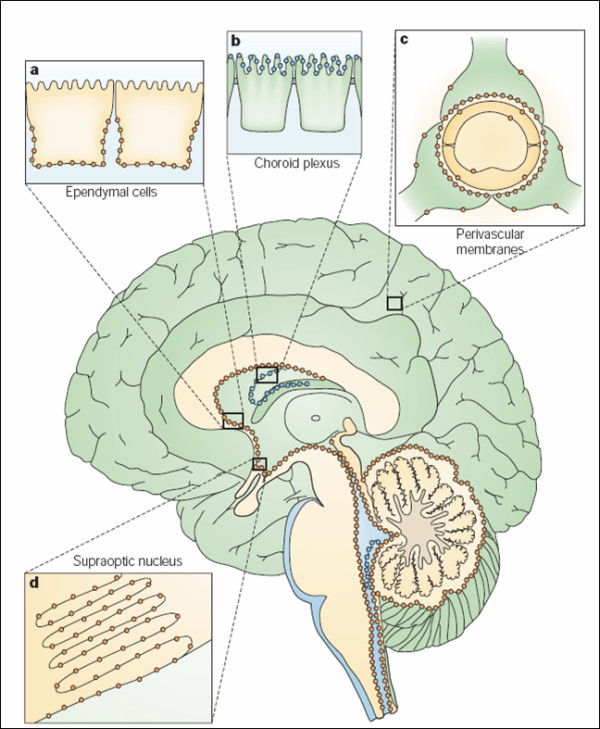
**Distribution in brain of aquaporin-1 (AQP1, blue) and AQP4 (orange), schematically illustrated on a sagittal section of a human brain**. a: AQP4 occurs in the basolateral membrane of ependymal cells. b: AQP1 is expressed at the apical membrane of choroid plexus epithelial cells. c: AQP4 is concentrated in astrocytic end-feet, specifically in those membrane domains that abut on brain capillaries or on pia. d: AQP4 is expressed in glial lamellae of the supraoptic nucleus and other osmosensitive regions. AQP4 also occurs in non end-feet membranes of astrocytes, but at comparatively low concentrations. In the neocortex, AQP4 expression in non end-feet membranes increases from deep to superficial layers. The cerebellum shows the opposite gradient, with higher concentrations in the granule cell layer than in the molecular layer. Reprinted with permission from [20].

**Figure 2:**
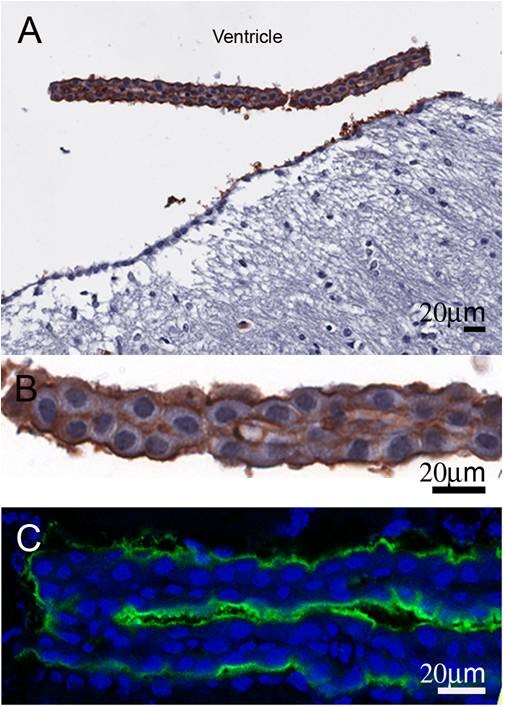
**AQP1 localisation in choroid plexus epithelial cell in wild-type mice**. Mouse coronal paraffin sections were incubated with rabbit anti-AQP1 antibody at 4°C overnight followed by incubation of anti-rabbit Ig conjugated with biotin and standard ABC technique (A and B) or anti-rabbit Ig conjugated with Alexa 488 (C). Positive staining for AQP1 is on the apical surface of the epithelial cells of normal wild-type mice. Panel B is the high magnification image of panel A. (Owler *et al*, unpublished data)

There are two main aquaporins within the CNS: AQP1 and 4 (Figure 1). AQP1 is found in the apical membrane of the choroid plexus [4,5] (Figure 2). It appears shortly after the choroid plexus in embryonic development [6] and is localised to the apical membrane. With ageing, choroidal AQP1 levels may be reduced [7]. AQP1 is also found in other organs such as red blood cells, salivary glands, cardiac muscle and kidneys. These findings appear consistent across species, but recently Arciénaga *et al*. [8] have demonstrated a more widespread distribution of AQP1 in non-human primates including white matter astrocytes, Schwann cells along oculomotor and trigeminal nerves as well as in neurons on the surface of pial blood vessels. AQP4 is located in the astrocyte foot processes that surround capillaries in the CNS as well as along the basolateral membrane of the ventricular ependymal cells. Its distribution appears consistent across species. A third aquaporin, AQP9, is also found within the CNS but is limited to small populations of catecholaminergic neurons as well as in astrocytes in cortical grey matter and hippocampus [8,9]. AQP9, being an aquaglyceroporin, also transports glycerol, lactate and other molecules [10,11]. It has been proposed to have a role in regulation of energy metabolism in the brain [12].

Naturally, the identification of a water channel protein in the apical membrane of the choroid plexus epithelium has generated interest in its role in CSF production, and consequently CSF circulation disorders. While AQP1 does appear to contribute to CSF production, its role in the pathophysiology of hydrocephalus has not been well studied and remains unclear. Studies demonstrating a role for AQP4 in relation to brain oedema in tumours, trauma and stroke as well as the observation that a small proportion of AQP4-null mice develop hydrocephalus, has also generated interest in the role of AQP4 in CSF circulation disorders.

There is no doubt that treatments for hydrocephalus and other CSF circulation disorders could be improved. Developing new technologies and improving CSF shunts and surgical procedures is important. However, more substantial improvements in the treatment of hydrocephalus are likely to be achieved by advancing knowledge of CSF physiology in health and disease [13]. Molecules such as aquaporins are an avenue of research through which the latter may be achieved. This review summarises current knowledge and concepts of CSF physiology and CSF circulation disorders as they relate to aquaporins. Strategies to further examine aquaporins in CSF circulation disorders are suggested.

## Aquaporin 1

### AQP1 and CSF production

**Figure 3:**
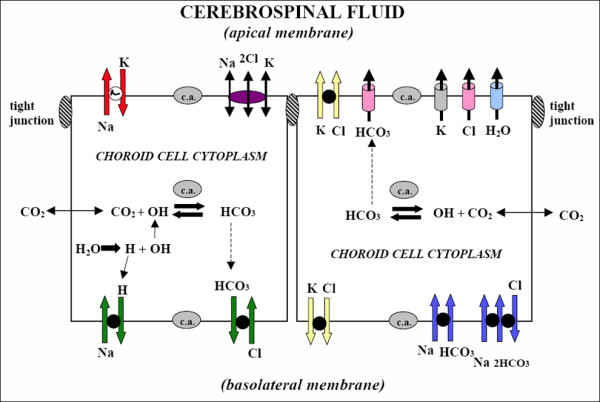
**Ion transporters and channels in mammalian choroidal epithelium**. CSF secretion results from coordinated transport of ions and water from basolateral membrane to cytoplasm, then sequentially across apical membrane into ventricles. On the plasma-facing membrane is parallel Na^+^-H^+ ^and Cl^-^-HCO_3_^- ^exchange bringing Na^+ ^and Cl^- ^into cells in exchange for H^+ ^and HCO_3_^-^, respectively. Also basolaterally located is Na^+^-HCO_3_^- ^cotransport (NBCn1) and Na-dependent Cl^-^-HCO_3_^- ^exchange that modulate pH and perhaps CSF formation. Apical Na^+ ^pumping maintains a low cell Na^+ ^that sets up a favorable basolateral gradient to drive Na^+ ^uptake. Na^+ ^is extruded into CSF mainly via the Na^+ ^pump and, under some conditions, the Na^+^-K^+^-2Cl^- ^cotransporter. K^+^-Cl^- ^cotransport helps maintain cell volume. Apical channels facilitate K^+^, Cl^- ^and HCO_3_^- ^diffusion into CSF. Aquaporin 1 (AQP1) channels on CSF-facing membrane mediate water flux into ventricles. Polarized distribution of carbonic anhydrase (c.a.) and Na^+^-K^+^-ATPase, and aquaporins, enable net ion and water translocation to CSF. Reprinted with permission from [14].

CSF production has choroidal and extrachoroidal components. Choroidal CSF secretion relies on the active production of an osmotic gradient. This osmotic gradient is driven mainly by carbonic anhydrase and Na^+^/K^+ ^ATPase. For a review of CSF production see Johanson *et al *(Figure 3) [14]. AQP1 in the apical membrane allows water to follow the osmotic gradient. AQP1 is important in maintaining this osmotic permeability of the apical membrane which is reduced by 4.8-fold in AQP1-null mice [15]. Only one study [15] has examined the role of AQP1 in CSF production and maintenance of intracranial pressure. In AQP1-null mice, CSF production was approximately 20% less than in wild-type mice (0.38 ± 0.02 vs. 0.30 ± 0.01 μl min^-1^). CSF pressure was also 56% lower in AQP1-null mice (9.5 ± 1.4 vs. 4.2 ± 0.4 cm H_2_O) which may have been contributed to, but was not fully explained by, a lower central venous pressure in these mice. While apical AQP1 is well placed to significantly contribute to CSF production, how the passage of water across the basolateral membrane is facilitated is not clear. There are reports of AQP1 being identified in the basolateral membrane of choroid plexus epithelium [5,16] but it is relatively scarce.

### AQP1 in hydrocephalus

Understanding the response of AQP1 to changes in CSF pressure and, in particular hydrocephalus, is important for determining a possible therapeutic potential. If AQP1 expression was to be significantly down-regulated in hydrocephalus, its therapeutic potential would be limited. However, if it remained unchanged or even were increased then pharmacological blockade might result in a therapeutically beneficial reduction in CSF production. Mao *et al*. [17] examined AQP1 in response to cisternal kaolin-induced hydrocephalus in the rat. In this chronic model of hydrocephalus, AQP1 protein and mRNA were unchanged compared to controls at 4 weeks and at 9 months after induction of hydrocephalus. However, in this study the choroid plexus was not studied separately; instead whole brain lysates were studied.

We have studied AQP1 expression and localisation in the choroid plexus of hydrocephalic adult mice using a cisternal kaolin injection model (Owler *et al*, unpublished data). We have found that AQP1 protein is unchanged compared to saline injected controls at 3 and 5 days post-injection of kaolin using Western Blot analysis of choroid plexus tissue. AQP1 mRNA levels were lower in hydrocephalic mice at 3 days post-kaolin injection but unchanged compared to saline-injected controls at 5 days post-kaolin. There is no clear explanation for this finding. In may be a reflection of an early and temporary reduction in AQP1 transcription in response to hydrocephalus. This needs to be clarified by further studies in other models of hydrocephalus.

**Figure 4:**
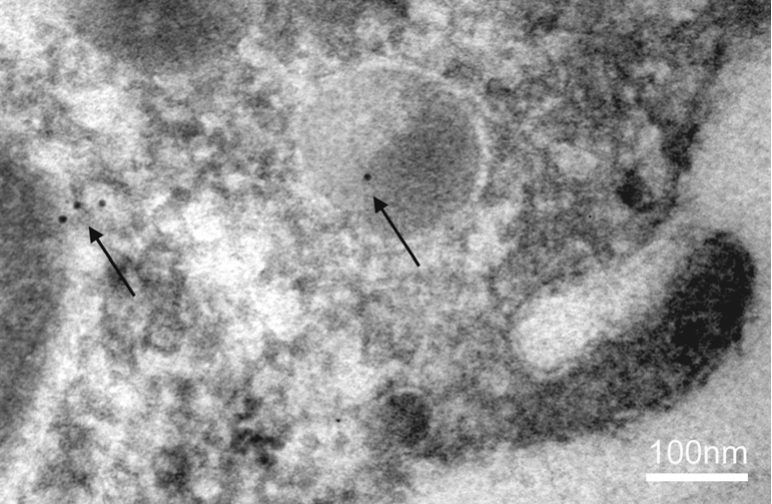
**Immuno-gold electron micrograph of choroid plexus epithelial cell from a wild type mouse after cisternal kaolin injection**. There is gold labelling (arrows) of AQP1 within the cytoplasm and a lysosome. (Owler *et al*, unpublished data).

**Figure 5:**
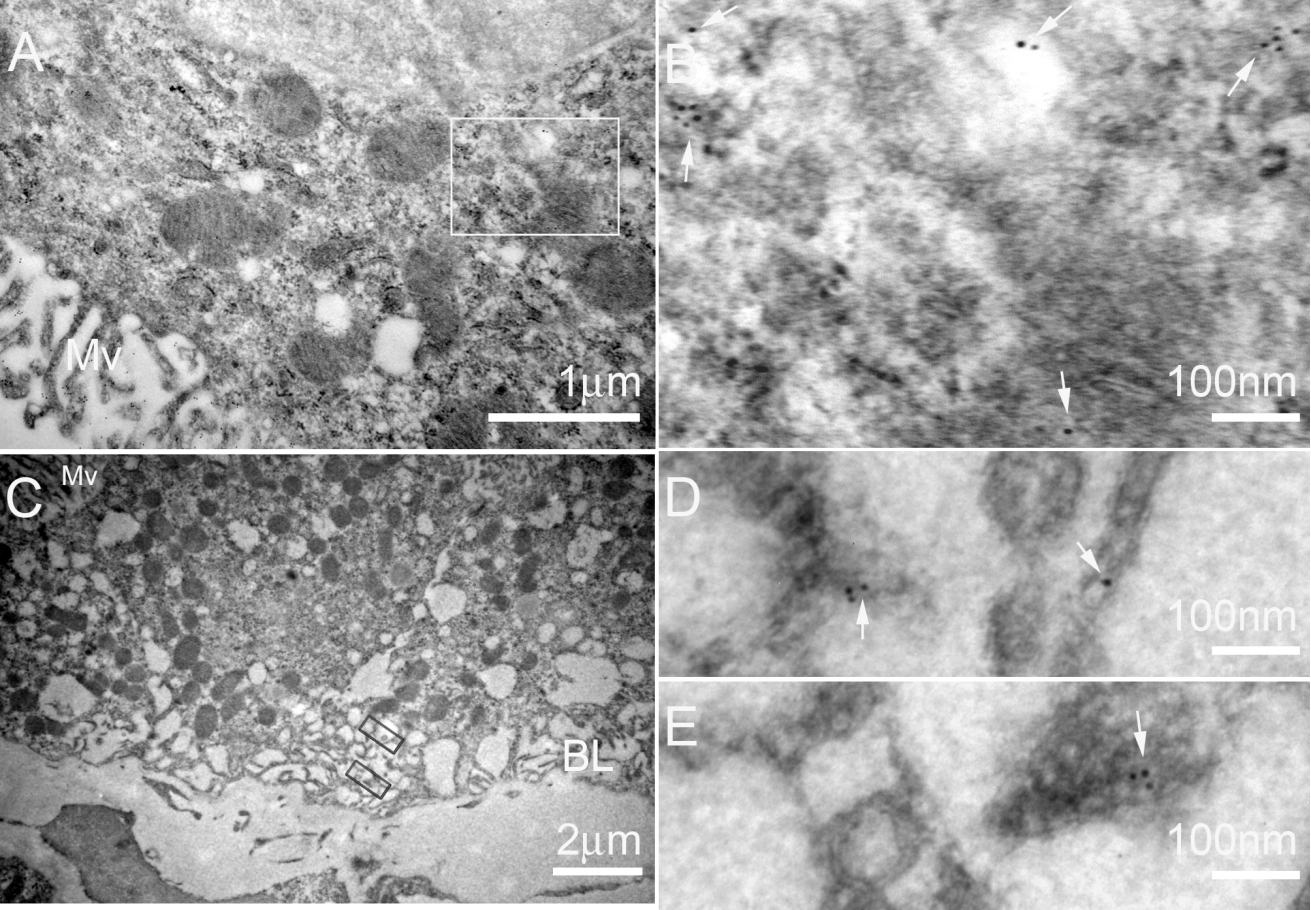
**Representative immuno-electron micrographs of choroidal epithelium in kaolin-injected hydrocephalic WT mice**. **A and C: **low magnification image of choroidal epithelium of hydrocephalic mice. **B, D and E: **Higher magnification electron micrographs corresponding to the boxes in A and C show gold particles (white arrows) in the cytoplasm (B) and in the basal membrane of the epithelia (D and E). Mv: microvilli; BL: basolateral membrane. (Owler *et al*, unpublished data).

While there appeared to be no change in the overall level of AQP 1 protein, immunohistochemistry staining for AQP 1 in choroid plexus specimens suggested that there was AQP1 staining in the cytoplasm of kaolin-injected mice compared to saline-injected controls. This was further studied using immuno-gold electron microscopy. We were able to confirm AQP1 gold labelling within the cytoplasm of choroid plexus epithelial cells which was absent in control mice (Figure 4). Furthermore this gold-labelling was also associated with endosomes and lysosomes. There was also gold-labelling of AQP1 along the basolateral membrane. The changes in AQP1 localisation were associated with other ultrastructural changes (Figure 5) reported by other authors in relation to the choroid plexus in hydrocephalus [18]. Such ultrastructural changes include a reduction in the number of microvilli which become shorter and swollen, the appearance in the cytoplasm of primary and secondary endosomes and lysosomes within the cytoplasm as well as the appearance of intercellular clefts. Despite the appearance of the latter, tight junctions at the apical membrane remained intact. Apoptosis was not present in our study or those of others [18]. The association of these changes, particularly those of the microvilli, with the observation that AQP1 localisation is altered raises the question of whether the changes in AQP1 localisation are a reflection of ultrastructural alterations or whether they reflect a compensatory mechanism for hydrocephalus, or both.

### Therapeutic potential of AQP1

**Figure 6:**
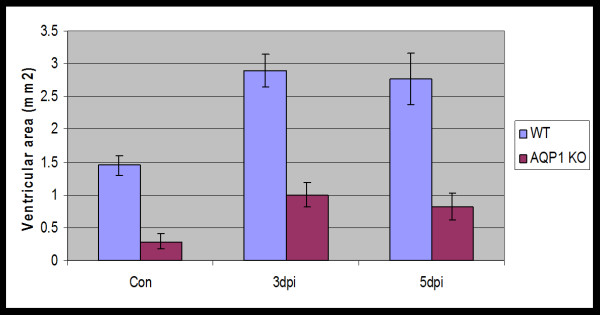
**Histograms representing mean ventricular areas of the wild-type (WT)(Blue) and AQP1-null mice (AQP1 KO)(Red) at 3 and 5 days post kaolin injection (dpi) compared to saline injected control (con) mice**. AQP1-null control mice (n = 14) had lower ventricular areas compared to wild-type control mice (n = 11). AQP1-null mice (n = 6 at 3 dpi; n = 11 at 5 dpi) did not demonstrate the same degree of ventricular dilation as wild-type mice (n = 8 at 3 dpi; n = 16 at 5 dpi) at either 3 or 5 days after cisternal kaolin injection. Data are means +/- SEM. (Owler et al, unpublished data).

The findings reported above may suggest that AQP1 is not a therapeutic target for management as AQP1 is internalised in response to hydrocephalus. Internalisation of AQP1 clearly renders it non-functional in terms of CSF production. However, we are of the opinion that AQP1 is still important as a possible therapeutic target for two reasons. Firstly, a large proportion of AQP1 remains in the apical membrane in response to hydrocephalus and therefore must still contribute to CSF production even in the face of hydrocephalus. Secondly, we found that AQP1-null mice do not develop hydrocephalus, or at least, the degree of ventricular dilation achieved in the cisternal kaolin-injection model, is much less than that in wild-type mice (Figure 6).

While there are some studies of AQP1 in experimental hydrocephalus, there is a paucity of human data in relation to aquaporins and hydrocephalus. Longatti *et al *[19] reported AQP1 immunostaining in nine choroid plexus tumours and found that staining patterns varied between individual tumours. Only in one case was there communicating hydrocephalus and this patient's tumour had strong AQP1 staining. We are currently in the process of accumulating choroid plexus specimens from patients with and without hydrocephalus to determine whether our observations in experimental hydrocephalus translate into the clinical environment.

### Regulation of AQP1

Regulation of AQP1 may occur through several mechanisms. First, the function may be altered by various compounds or biological processes. Second, because the function of AQP1 is dependent on its location in the apical membrane of the choroid plexus, AQP1 may be regulated by changes in its location within the cell. Finally, the overall level of AQP1 protein may be regulated in response to various physiological or pathophysiological parameters. There is some evidence for each of these mechanisms although the details remain unclear. In relation to regulation of AQP1 function and permeability, it is well known that mercurial compounds inhibit AQP1 [20]. The human binding site for Hg^2+ ^is Cys189 [21]. Phosphorylation of AQP1 may influence AQP1 function and water permeability [22]. Phosphorylation of different amino acid residues may result in changes in increased water permeability in response to vasopressin and which is blocked by atrial natriuretic peptide [23]. In terms of the overall level of AQP1 protein, there is some evidence that regulation of AQP1 in the choroid plexus does occur. Spontaneously hypertensive rats demonstrate increased CSF secretion and more rapid CSF turnover [24]. This enhanced CSF secretion appears to be facilitated by an up-regulation of AQP1 in the choroid plexus. This up-regulation of choroidal AQP1 was recently reported by Tomassoni *et al*. [25] although the mechanism underlying this up-regulation again remains unclear.

Dexamethasone has been found to increase expression of AQP1 in the lungs of rats [26]. Kobayashi *et al*. [27] also reported that AQP1 expression was increased by glucocorticoid when applied to cultured brain endothelial cells. However the effects of steroids on the expression of AQP1 in the choroid plexus have not yet been tested. It does however raise an interesting question particularly in relation to steroids as an aetiology of pseudotumor cerebri [28].

Degradation of AQP1 probably occurs through AQP1 ubiquitination. This refers to the post-translational modification of a protein associated with the attachment of a small protein, ubiquitin, which labels a protein for degradation. AQP1 ubiquitination appears to be dependent on tonicity of the local cellular environment. This was shown by Leitch *et al*. [29] who reported that AQP1 ubiquitination was reduced and AQP1 half-life increased in response to hypertonic stress in fibroblast cell culture.

In some tissues there is evidence to suggest that AQP1 can be regulated though translocation between the cytoplasm and apical membrane. Page *et al*. [30] described the movement of AQP1 between the cytoplasm and plasma membrane associated with caveolae in rat cardiac myocytes in response to osmotic changes. In cholangiocytes, Tietz *et al*. [31,32] found that AQP1 moves to the apical cell membrane from intracellular vesicles in response to choleretic agonists to facilitate bile secretion. Similar findings were also reported by Marinelli *et al*. [33-35]. Our finding that choroidal AQP1 is internalised after induction of hydrocephalus secondary to kaolin injection in the mouse also supports this notion and we speculate that this may be a form of compensatory response to hydrocephalus.

The mechanisms underlying this translocation or trafficking of AQP1 have been studied by Conner *et al*. [36] in cell culture using HEK293 cells. That group concluded that AQP1 trafficking was regulated by the tonicity of the cellular environment and that this was mediated through a protein kinase C-dependent mechanism and that the trafficking itself was dependent on microtubules. Further studies to determine mechanisms of AQP1 regulation are needed and may present an opportunity to direct therapy.

## Aquaporin 4

### Location and function

**Figure 7:**
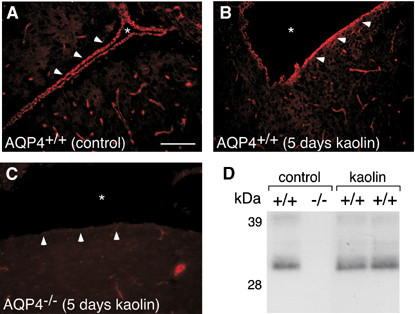
**Aquaporin-4 protein expression in mouse model of hydrocephalus**. Aquaporin-4 immunostaining of AQP4^+/+ ^control mouse (**A**), AQP4^+/+ ^(**B**), and AQP4^-/- ^(**C**) mice 5 days after kaolin injection. Asterisks indicate lateral ventricle, white arrowheads indicate ventricular ependyma. **(D) **Immunoblot of AQP4 protein expression in AQP4^+/+ ^and AQP4^-/- ^control mice and AQP4^+/+ ^mice at 5 days after kaolin injection. Scale bar = 100 *μ*m. Reprinted with permission from [60].

AQP4 is located primarily in astrocytic end-feet, the external and internal glial limiting membranes and the basolateral membrane of ependymal cells [37,38] (Figure 7). Shen *et al*. [39] noted that the AQP4 expression was absent in 1 day-old non hydrocephalic H-Tx rats. AQP4 expression was noted in the cerebral cortex and ependyma at 1 week of age and then was also seen in the sub-pial zones of the cortex, periventricular regions and the perivascular foot processes of astrocytes at 4 and 8 weeks of age. These authors suggested that this change in AQP4 expression reflected the changes in CSF circulation that occurs in neonates.

Localisation to the end-feet of astrocytes is dependent on interaction with the dystrophin complex including alpha-syntrophin [40]. Its primary role appears to be in regulating the distribution of water through the brain. It is co-localised with the inwardly-rectified potassium channel, Kir4.1. It is thus important in the spatial buffering of potassium and therefore neuronal excitability. Binder *et al*. [41] reported AQP4-null mice had an increased seizure threshold compared to wild-type mice. In contrast, seizures were more severe in alpha-syntrophin-deficient mice in which AQP4 is no longer concentrated in the astrocyte end-feet [42].

There has been some argument regarding the importance of AQP4 in maintaining integrity of the blood-brain barrier (BBB). Nicchia *et al*. [43] questioned the role of AQP4 in maintaining the BBB after studying *mdx *mice with a marked reduction in dystrophin expression, which is important for AQP4 localisation to the astrocyte end-feet. Zhou *et al*. [44] reported marked abnormalities of the blood brain barrier in AQP4-null mice as well as other abnormalities such as altered astrocyte GFAP expression. Saadoun *et al*. [45] using a variety of techniques in AQP4 -mice concluded that there were no structural alterations of the brain due to AQP4 deletion including the BBB. Several previous publications of the same group also suggested no alteration of the BBB of AQP4 mice from that lab [46-48].The latter group also examined the BBB of the Nanjing AQP4-null mice of the type used in the study of Zhou *et al*. and again found no alterations in the BBB. A number of technical artefacts were suggested as being responsible for the reported disruption of the BBB in the report of Zhou *et al*.

AQP4 is the most abundant and widespread aquaporin in the brain and occurs in a short isoform (301 amino acids; M23) and a long isoform (323 amino acids; M1)[49]. AQP4 forms heterotetramers and is a component of orthogonal arrays of particles (OAPs) [38]. These OAPs are intramembrane structures visible on freeze-fracture preparations. The AQP4 M23 isoform promotes formation of OAPs in contrast to AQP4 M1 isoform which does not form OAPs without AQP4 M23. The ratio of AQP4 M23: AQP4 M1 isoforms will therefore influence the formation of OAPs, and as OAPs promote cell-cell adhesion the isoform ratio may also influence AQP4 function [50-52].

The role of AQP4 in handling brain water and therefore brain oedema has been demonstrated in several pathologies including cerebral tumours, traumatic brain injury, cerebral ischaemia or stroke and brain abscess [53]. In AQP4-null mice, after induction of conditions in which vasogenic oedema forms such as around cerebral tumours and in traumatic brain injury [47], there is increased brain oedema. However, in other conditions such as cerebral ischaemia where cytotoxic oedema predominates, AQP4-null mice demonstrate a reduction in brain oedema [46]. AQP4 is also upregulated by conditions that induce oedema such as cerebral tumours [54,55] or cerebral infarction [56]. There is a report of AQP4 staining in the choroid plexus epithelium of rats although staining was diffuse throughout the cytoplasm and not detected on the apical or basolateral membranes. The significance of this finding is not clear [57].

### AQP4 in hydrocephalus

There are some conflicting reports in the AQP4-null phenotype in relation to ventricular size and CSF dynamics. Manley *et al*. [46] reported no difference in the brains of AQP4-null and wild-type mice, in addition intracranial pressure and compliance were unaltered. However, Li *et al*. [58] reported that the majority of AQP4-null mice demonstrated smaller ventricular sizes, reduced CSF production and increased brain water content compared to wild-type mice. These authors also concluded that AQP4 was important in maintaining the ependymal integrity in mice. Akin to the debate regarding the AQP4 and the BBB, Saadoun *et al*. [45] reported no changes in ventricular volume or anatomical features of these AQP4-null mice both from the San Francisco and Nanjing laboratories.

The main role of AQP4 in hydrocephalus appears to be as a compensatory mechanism. Mao *et al*. [17] reported an increased AQP4 mRNA in rats 4 weeks and 9 months after induction of hydrocephalus secondary to kaolin injection although no differences in AQP4 protein could be detected by Western blot analysis. Shen *et al*. [39] found that AQP4 expression was markedly increased in 8 week-old H-Tx rats with spontaneously arrested hydrocephalus compared to non-hydrocephalic H-Tx rats. The authors suggested that the increase in AQP4 reflected the development of a compensatory/alternative pathway for CSF resorption as is thought to occur in arrested hydrocephalus.

Using a communicating model of hydrocephalus secondary to subarachnoid inflammation after intraparenchymal injection of L-α-lysophosphatidylcholine (LPC) stearoyl, Tourdias *et al*. [59] also reported increased expression of AQP4 using quantitative immunohistochemistry. Initially AQP 4 expression appeared confined to astrocytic end-feet but at later time points astrocytes became hypertrophied and AQP4 expression appeared throughout the extent of the plasma membrane. Using magnetic resonance diffusion-weighted imaging, the degree of experimental hydrocephalus was correlated with up-regulation of AQP4 and the periventricular apparent diffusion co-efficient. The authors concluded that the pattern of AQP4 expression was consistent with an adaptive response to the hydrocephalus.

The finding of increased AQP4 expression in various models of hydrocephalus has been interpreted as a compensatory mechanism to allow for transependymal/parenchymal CSF absorption. Such adaption may be a mechanism that allows for the development of compensated or arrested hydrocephalus. This hypothesis was tested by Bloch *et al*. [60] who studied the development of hydrocephalus after cisternal kaolin injection in AQP4-null mice (Figure 7). The degree of ventricular dilation and intracranial pressure change were both significantly greater in AQP4 knockout mice compared to wild-type mice at 3 and 5 days after kaolin injection. Furthermore there was a 2-3% increase in brain parenchymal water content in both wild-type and AQP4-null mice at 3 days post-injection compared to baseline, although there was no difference between wild-type and AQP4-null mice. This increase was interpreted as evidence that hydrocephalus caused an increase in intraparenchymal water. These authors also discussed the relative importance of astrocytic versus ependymal AQP4 in hydrocephalus. According to their modelling, AQP4 associated with the astrocytic end feet was more important than that associated with the ependyma. It should be noted that the ependyma is frequently disrupted in hydrocephalus, particularly in the frontal and occipital horns where shear strains are greatest [61].

### Spontaneous hydrocephalus in AQP4-null mice

There is a small but consistent rate of spontaneous hydrocephalus in AQP4-null mice that has been reported by several groups. Feng *et al*. [48] reported a 9.6% incidence of spontaneous hydrocephalus secondary to aqueduct stenosis. The hydrocephalus was severe with raised intracranial pressure resulting in death within six weeks. Li *et al*. [58]found that in a minority of AQP4-null mice (7%) the aqueduct was narrowed and there was enlargement of the lateral ventricles in contrast to the other 93% that they reported to have small lateral ventricles in comparison to wild-type mice (see above).

According to Feng *et al*. [48], mice that had developed hydrocephalus secondary to aqueduct stenosis displayed marked disorganisation of the ependyma at the aqueduct. Similar findings were reported by Li *et al*. [58]. Regional ependymal disorganisation was also seen in some non-hydrocephalic mice in other areas and ependymal disorganisation was also seen in heterozygotes. Wild-type mice displayed no such regions. Whether mutations in or absence of AQP4 in the ependyma of hydrocephalic patients with sporadic aqueduct stenosis have a role in pathogenesis, is yet to be tested.

### AQP4 regulation

The regulation of AQP4 may be categorised in a similar manner to that of AQP1 and was recently reviewed by Yukutake and Yasui [62] and previously by Gunnarson *et al*. [22]. AQP4 has been considered to be insensitive to mercury and this has been demonstrated by a number of studies [63-66]. However, Yukutake *et al*. [67] recently demonstrated that the water permeability of AQP4 M23 isoform was reversibly decreased in response to mercury using AQP4 M23-proteoliposomes in a stopped-flow analysis. The permeability of AQP4 may also be altered through phosphorylation. Phosphorylation of Ser180 decreases water permeability of AQP4 [68] while phosphorylation of Ser111 increases water permeability [69,70]). The mechanism underlying phosphorylation and how it relates to physiological function is unclear at present.

There is also some evidence for regulation of the overall level of AQP4. As for AQP1, the tonicity of cellular environment may be important for AQP4. Zeng *et al*. [71] demonstrated that AQP4 was down-regulated in response to treatment with intravenous 10% hypertonic saline in a rat model of ischaemia. AQP4 protein levels are increased in response to various pathological processes. In the spontaneously hypertensive rat, AQP4 levels were increased in the frontal cortex, striatum and hippocampus between 4 and 6 months of age which coincides with the development hypertension and associated pathological changes in these animals [25]. Interestingly, Koyama and Tanaka [72] also recently demonstrated that ET-1, a selective ET_B _receptor agonist and vasoconstrictor peptide, resulted in down-regulation of AQP4 in rat brain.

Localisation of AQP4, particularly in the astrocytic end feet is important for function as discussed above. A recent study suggests that trafficking of AQP4 may be used as a regulatory mechanism for AQP4. Moeller *et al*. [73] found that vasopressin results in internalisation (to the cell cytoplasm) of AQP4 in *Xenopus laevis *oocytes which is mediated by the V1_a _receptor. This resulted in decreased water permeability. The effect was reduced with mutation of Ser^180^; phosphorylation which has already been reported to decrease water permeability [68]. This phosphorylation was mediated by protein kinase C.

## Pharmacological modulation of AQP1 and AQP4

Determining the importance of AQP1 and 4 in hydrocephalus and CSF production would be facilitated by the availability of a non-toxic specific AQP1 or 4 blocking agents. Identifying such an AQP1 blocker remains a challenge [74]. It is known that AQP1 is blocked by Hg^2+ ^and other mercurial compounds but these are toxic [75]. As mentioned, AQP4 has been considered insensitive to Hg^2+ ^[76]. However more recently, Yukutake *et al*. [67] have suggested that the water permeability of the M23 isoform may be reduced by Hg^2+^. Tetraethylammonium (TEA) was also suggested as an AQP1 inhibitor [77] although the efficacy of TEA is likely to be minimal [78]. Corticosteroids have been reported to up-regulate AQP 1 in the rat lung [26], sheep fetal lung [79] as well as other tissues [80,81]. There is as yet, no evidence that steroids influence choroidal AQP1. AQP4 expression is not altered by corticosteroids [74,82].

Arylsulfonamides, including acetazolamide, a carbonic anhydrase inhibitor that is commonly used to reduce CSF production in the management of pseudotumor cerebri and other clinical situations, have been suggested as pharmacological blockers of AQP1 and 4. This has generated considerable controversy. Ma *et al*. [83] reported that acetazolamide reduced osmotic permeability via interaction with AQP1. Osmotic permeability was assessed using a swelling assay of *Xenopus laevis *oocytes expressing AQP1, and the same group reported similar findings in a swelling assay using human embryonic kidney (HEK292) cells expressing pEGFP/AQP1 [84]. Using the oocyte swelling assay, Huber *et al*. [85] reported that acetazolamide and several other compounds [86,87] had some inhibitory affect on water permeability of AQP4. The oocyte swelling assay technique has been criticised, and using a stopped-flow light-scattering water permeability assay and a marker dilution technique Yang *et al*. found no evidence that acetazolamide inhibits AQP1 or 4 [78,88]. In addition, using modifications of the *Xenopus laevis *oocyte osmotic permeability assay, Sorgaard & Zeuthen [89] found no effect of acetazolamide on AQP1. Recently Tanimura *et al*. [90] who also used a stopped-flow analysis technique, reported a reversible reduction in water permeability of AQP4 due to acetazolamide. The issue appears to remain unresolved with Huber and colleagues [86,87,91] continuing to argue in favour of an inhibitory effect of acetazolamide on AQP1 and 4.

A small inhibitory effect of bumetamide, a loop diuretic that blocks the Na-K-Cl co-transporter, on AQP4 water permeability [92] has lead to the development of other related molecules based on the structure of bumetamide. The arylsulfonamide AqB013 which has been reported by Yool *et al*. [93] to block both AQP1 and 4 is such an example although its therapeutic potential remains to be tested.

## Other therapeutic strategies for AQP1 modulation

Apart from pharmacological blockade of AQP1, there are several other potential routes to modulation of AQP 1 expression, none of which have thus far been explored. These include methods to increase AQP1 degradation or reduction of AQP1 expression/transcription. Although our knowledge of the mechanisms and pathways underlying choroidal AQP1 regulation is lacking there are possible techniques that may be employed to increase AQP1 degradation. This includes the intraventricular administration of AQP1 antibodies which could potentially result in AQP1 internalisation and degradation, thus temporarily reducing CSF production. Further understanding the mechanisms through which AQP1 is internalised, as we have observed in our studies of choroidal AQP1 in hydrocephalic mice, may provide additional useful information to allow these pathways to be manipulated.

**Figure 8:**
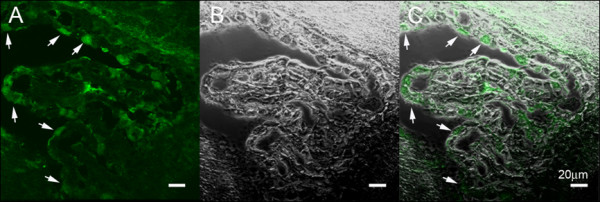
**Lentiviral-GFP localisation in choroid plexus epithelial cells in mice**. Normal Quackenbush Swiss mice received intraventricular injection of lentiviral-GFP (10^7 ^virus genomes in 10 μL of sterile saline) and harvested after 7 days of injection. Frozen coronal section of the brain were incubated with rabbit GFP antibody at 4°C overnight followed by incubation of anti-rabbit Ig conjugated with Alexa 488. GFP signals were found in the cytoplasm of some choroid epithelial cells (arrows) in lateral choroid plexus in panel A (confocal image) and C (composite of A and B). Panel B is the phase contrast image of the same field in A. (Owler *et al*., unpublished data).

AQP1 knockdown *in vivo*, or gene therapy, is another potential tool for modulating AQP1 and thus CSF production. A small interfering RNA (siRNA) against AQP1 has been used by Boassa *et al*. [94] to study AQP1 function in choroid plexus cell culture. The use of si or shRNA, delivered though lentiviral or other vectors to transfect choroid plexus epithelial cells to reduce AQP1 expression may be feasible (Figure 8) [95]. Splinter *et al*. [96] has used AQP1 siRNA to reduce AQP1 expression in isolated intrahepatic bile duct units in the rat. This resulted in reduced water transport in these bile duct units in response to both an osmotic challenge and a secretory agonist. Kim *et al*. [97] reported that TTF-1 (thyroid transcription factor -1) increased *AQP1 *transcription. The 5' region of *AQP1 *has multiple binding sites for TTF-1 the expression of which is also seen in the choroid plexus. Intraventricular injection of antisense TTF-1 oligodeoxynucleotide in rats resulted in a reduction of AQP1 mRNA and protein in the choroid plexus. These rats also had an increased survival compared to controls after water intoxification.

## Conclusions

Aquaporin 1 makes a substantial contribution to CSF production and is a potential therapeutic target in the management of CSF circulation disorders. Aquaporin 4 is important in brain water homoeostasis and consequentially in conditions involving both cytotoxic and vasogenic oedema. In hydrocephalus AQP4 has a protective effect by allowing resorption of transependymal CSF into brain capillaries. There is considerable scope for improving our understanding of aquaporins in relation to CSF physiology in health and in diseases such as hydrocephalus. The ultimate potential of aquaporin modulators in the management of these conditions and others remains to be determined. Continued study of aquaporins in hydrocephalus and other conditions is needed.

## Competing interests

The authors declare that they have no competing interests.

## Authors' contributions

All authors contributed to the writing of this review. All authors have read and approved the final version of the manuscript.
